# Prolonged expression of the γ-H2AX DNA repair biomarker correlates with excess acute and chronic toxicity from radiotherapy treatment

**DOI:** 10.1002/ijc.25953

**Published:** 2011-02-03

**Authors:** Emma C Bourton, Piers N Plowman, Daniel Smith, Colin F Arlett, Christopher N Parris

**Affiliations:** 1 Brunel Institute of Cancer Genetics and Pharmacogenomics, Division of Biosciences, Brunel UniversityUxbridge, Middlesex, United Kingdom; 2 Department of Radiotherapy, St. Bartholomew's HospitalWest Smithfield, London, United Kingdom; 3 Genome Damage and Stability Centre, Science Park Road, University of SussexFalmer, Brighton, Sussex, United Kingdom

**Keywords:** normal tissue toxicity, radiotherapy, predictive testing, DNA repair, γ-H2AX

## Abstract

The normal tissue tolerance levels to fractionated radiotherapy have been appreciated by a century of careful clinical observations and radiobiological studies in animals. During clinical fractionated radiotherapy, these normal tissue tolerance levels are respected, and severe sequelae of radiotherapy are avoided in the majority of patients. Notwithstanding, a minority of patients experience unexpectedly severe normal tissue reactions. The ability to predict which patients might form this minority would be important. We have conducted a study to develop a rapid and reliable diagnostic test to predict excessive normal tissue toxicity (NTT) in radiotherapy patients. A flow cytometric immunocytochemical assay was used to measure DNA damage in peripheral blood lymphocytes (PBL) from cancer patients exposed to 2-Gy gamma radiation. DNA damage and repair was measured by induction of cellular γ-H2AX in unirradiated and exposed cells at specific time points following exposure. In 12 cancer patients that experienced severe atypical NTT following radiotherapy, there was a failure to repair DNA double-strand breaks (DSB) as measured by γ-H2AX induction and persistence. In ten cancer patients that experienced little or no NTT and in seven normal (noncancer controls), efficient repair of DNA DSB was observed in the γ-H2AX assay. We conclude that a flow cytometric assay based on γ-H2AX induction in PBL of radiotherapy patients may represent a robust, rapid and reliable biomarker to predict NTT during radiotherapy. Further research is required with a larger patient cohort to validate this important study.

Radiotherapy (RT) is an important form of treatment of both local and regional disease with ∼50–60% of cancer patients receiving radiotherapy at some stage, either as monotherapy or in combination with surgery, chemotherapy and/or hormonal therapy.^1^ Improvements in tumour imaging, targeting of radiotherapy and optimisation of delivery schedules have led to significant improvements in tumour response and outcomes, and reduction in normal tissue toxicity (NTT).^2^ Nonetheless, NTT remains a limiting factor in the treatment of cancer.^3^

There is interpatient heterogeneity in acute and delayed NTT during and after radiotherapy, a phenomenon that has been observed for more than half a century.^4^ NTT has been graded by the Radiation Therapy Oncology Group (RTOG) into a standardised scale of acute and late responses for all tissue types.^3^ High-dose radical RT prescriptions risk higher levels of NTT, but it has long been appreciated that a small minority of patients manifest an excessive reaction even to low radiotherapy doses.^5^–^7^ Such “overreactors” (ORs) may respond so extremely to radiotherapy that the side effects are potentially lethal. Individuals with genetic defects in the repair of ionising radiation-induced DNA double-stranded breaks (DSB) are ORs, the classical example of such disease being ataxia telangiectasia (A-T). Patients with A-T have extreme clinical and cellular hypersensitivity to ionising radiation, suffering neurodegeneration and increased cancer incidence.^8^, ^9^ These individuals are usually diagnosed early in life because of the occurrence of recognisable clinical symptoms, and if requiring anticancer therapy will be treated in such a way to minimise life-threatening toxicity. However, asymptomatic and undiagnosed human DNA repair defects may leave individuals with a predisposition to cancer and an increased risk of overreacting during treatment. Such has been the case with four paediatric patients at St. Bartholomew's Hospital, London (three with lymphoma and one with angiosarcoma), which resulted in fatal clinical responses to radiotherapy.^10^, ^11^

RT primarily exerts a cytotoxic effect by causing DNA DSB in cells.^12^ The extent of normal cell damage and its repair governs the degree of NTT experienced by the patient. Most human cells have complex cellular DNA repair mechanisms that can reverse the effects of DNA damage induced by ionising radiation; these are physiological mechanisms to increase cell survival and maintain genomic stability. Therefore, the efficiency of DNA repair in cancer and normal cells is likely to play an important role in both tumour response and level of NTT experienced.

The desire to predict how individual patients respond to RT has been a goal of clinical and experimental oncology for many decades. A number of *in vitro* methods have been examined for their potential in predicting ionising radiation sensitivity. These include clonogenic cell survival assays,^7^ alkaline comet assays^13^ and micronucleus assays.^14^ In addition, attempts have been made to correlate the activity and expression of specific DNA repair genes and normal tissue response using a variety of molecular methods.^15^ These methods have been informative in defining genotoxic responses to radiation and a variety of DNA-damaging agents, but have been of limited use and potential as a rapid and technically simple test to be applied in the clinical setting, particularly prospectively, as they can take weeks or months to perform. Therefore, an assay that can provide results within a short time period before commencing RT is highly desirable.

To address this problem, we developed a flow cytometry assay of γ-H2AX as a predictive/clinical test to determine patient radiosensitivity. An initial step in the cellular repair of DNA DSB is the phosphorylation of the minor histone H2AX protein to form the γ-H2AX protein.^16^ Many thousands of γ-H2AX molecules accumulate at sites of DSB to form discrete nuclear “foci”, which can be visualised and quantified by a number of methods including *in situ* immunocytochemistry or flow cytometry. The number of foci is indicative of DNA DSB.^17^ Normally, with the passage of time (and over a predictable time in the presence of normal physiological repair mechanisms), the level of γ-H2AX diminishes as repair is completed; persistence of γ-H2AX indicates impaired DNA repair.

Our study aimed to examine whether there were significant differences in γ-H2AX depletion in irradiated peripheral blood lymphocytes (PBL) between patients who had developed excessive NTT from radiotherapy and control groups.

## Material and Methods

### Subject selection

Three groups of subjects were recruited to our study, which had been approved by local NHS ethics committee. Twelve patients who had experienced severe atypical NTT as a consequence of earlier RT were identified from follow-up clinics, forming one group; ten patients who had experienced little or no NTT acted as one control group; seven healthy, noncancer individuals comprised a second control group. Patient details including tumour histology and stage, radiotherapy dose and level of NTT experienced measured on the relevant RTOG scale are shown in Table 1. None of the patients selected for the study had received prior chemotherapy.

**Table 1:** Clinical details for all cancer patients (NOR and OR) used in this study

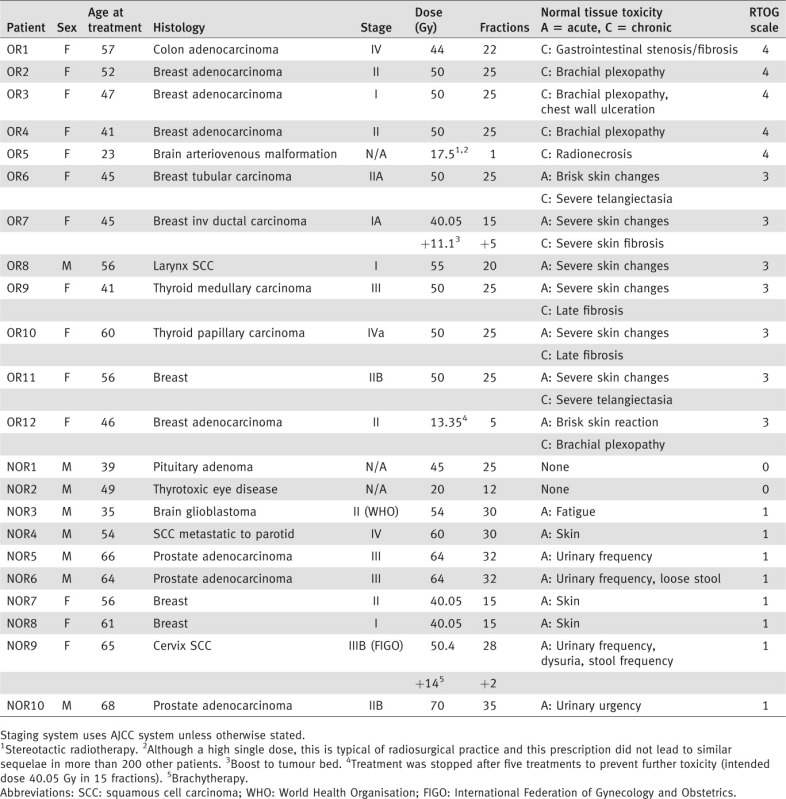

All blood samples were obtained by the clinical team with full patient informed consent. The testing process was performed by the laboratory team who were blinded to the identity of the patients. Limitations of ethical approval dictated that only one blood sample be taken from each patient; however, the number of PBL produced allowed testing of each sample on at least two separate occasions.

### Isolation of PBL

A 10-ml venous draw was taken from the patient's arm into heparinised containers, which were transported within 1 hr to Brunel University (Institute of Cancer Genetics and Pharmacogenomics). PBL were isolated by density centrifugation over Ficoll-Hypaque lymphocyte separation medium (LSM) (Fisher Scientific, Leicestershire, UK) according to the standard methods. In brief, 10 ml of blood was diluted 1:1 with RPMI 1640 medium (Fisher Scientific) and was carefully laid on to an equal volume of LSM and subjected to 20-min centrifugation at 400*g* in a table top centrifuge (Heraeus GmbH, Hanau, Germany). The buffy coat containing the PBL was carefully removed from the interface of the LSM and the blood serum and washed by a further centrifugation in 20-ml serum-free RPMI 1640 medium at 450*g* in a table top centrifuge. The number of PBL in each sample was determined with a haemocytometer; 10^7^ cells were frozen in liquid nitrogen for future use, and ∼4 × 10^6^ cells were set up in a T75 cell culture flask (Nunc, Fisher Scientific) in RPMI 1640 medium containing 10% foetal calf serum, 2 mM l-glutamine and 100 U/ml penicillin and streptomycin (Fisher Scientific). Cells were incubated in a humidified atmosphere at 37°C with 5% CO_2_ in air before gamma irradiation and staining for cellular γ-H2AX expression.

### Irradiation of PBL

PBL were irradiated in batches of four to five samples, according to the attendance of patients at clinic. Following the removal of 10^6^ PBL (untreated control), the remaining cells were irradiated to a dose of 2-Gy using gamma radiation from a ^60^Cobalt source (Puridec Technologies, Oxfordshire, UK) sited at a distance of 25 cm with a dose rate between 1.3 and 1.4 Gy per minute. A total of 10^6^ cells were harvested from the flask after 30 min, 5 hr and 24 hr for flow cytometric analysis of γ-H2AX induction.

### DNA damage dose–response curve

To determine the level of fluorescence induced by different doses of gamma radiation, and thus the level of γ-H2AX induction and DNA damage, PBL from a normal noncancer individual were irradiated with 0-, 2-, 4-, 6- and 8-Gy gamma irradiation and subjected to flow cytometric analysis. Cells were fixed and stained as described below.

### Immunocytochemical detection of γ-H2AX in PBL

In brief, untreated control cells and those treated with 2-Gy gamma radiation were pelleted by centrifugation and washed twice in phosphate-buffered saline (PBS) (Sigma Chemicals, Dorset, UK) before fixing in methanol/acetone (50:50% v/v) for 5 min at 4°C. Following three washes in PBS, mouse monoclonal anti-γ-H2AX (Millipore UK, Hampshire, UK) at 1:10,000 dilution was added to the cells for 1 hr at room temperature, followed by washing in PBS. Two hundred microliters of Alexa Fluor® 488 goat anti-mouse IgG (Invitrogen UK, Paisley, UK) diluted 1:1,000 was then applied to each sample for 1 hr at room temperature in the dark followed by counterstaining with 1 μg/ml propidium iodide. Cells were stored for not more than 24 hr before the commencement of flow cytometry. The level of fluorescence in control and irradiated cells was estimated by counting a minimum of 20,000 cells at each time point using an Epics XL-MCL flow cytometer (Beckman-Coulter, Berkshire, UK). The fluorescence in cells postirradiation was compared to the level of fluorescence in untreated control cells to provide a measure of relative fluorescence.

### Statistical analysis

Differences in the relative fluorescence in the PBL between patient groups were compared using a Student's unpaired *t*-test, specifically, γ-H2AX retention in PBL after 24 hr.

## Results

### Induction of fluorescence with escalating gamma radiation dose

**Figure 1:**
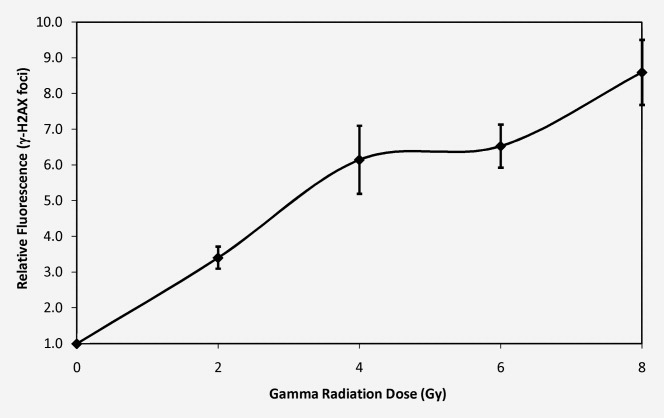
The induction of fluorescence in PBL derived from a normal (noncancer) individual. The level of fluorescence (γ-H2AX induction) increases with dose of gamma radiation by ∼1.5- to 2-fold per 2 Gy of gamma radiation. Error bars represent standard error of the mean fluorescence levels derived from at least two independent experiments. For all further experiments using patient samples, a dose of 2-Gy gamma radiation was used.

This experiment was essential to determine the dynamic range of fluorescence induction with ionising radiation and to determine an appropriate dose of radiation to use for future analyses with patient samples. Following the exposure of PBL from a normal individual to a range of gamma radiation doses, an ∼1.5- to 2-fold increment of relative fluorescence was observed with each dose (Fig. 1). The result of this dose–response experiment confirmed that future experiments would be conducted by exposing the samples to 2-Gy gamma radiation, an appropriate dose because most radical radiotherapy protocols are administered in a fractionated regimen of 2-Gy per dose.

### Determination of γ-H2AX induction in patient PBL

Twenty-nine blood samples were analysed in the flow cytometric assay: 12 from OR patients who had experienced severe atypical NTT after radiotherapy, ten from “non-overreactor” (NOR) patients who had experienced little or no NTT and seven from normal noncancer individuals (N) who had not received prior irradiation.

**Figure 2:**
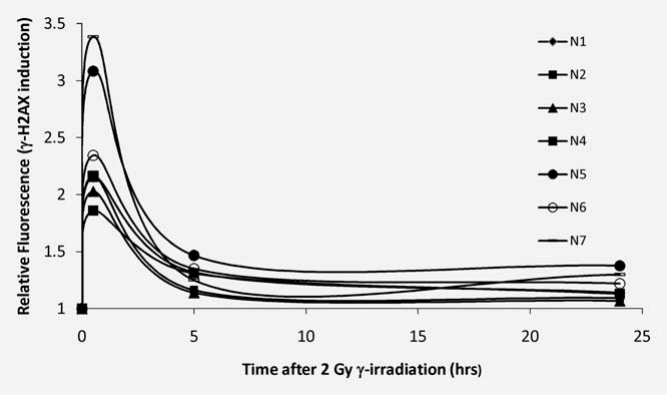
The results derived from the flow cytometric analysis of γ-H2AX induction from seven normal noncancer patients (normal: N1–7). A rapid rise in radiation-induced DNA DSB is revealed by an increase in relative fluorescence 30 min postexposure. This is followed by a steady decline in fluorescence, due to repair of DNA DSB and at 24 hr fluorescence levels have returned to near unirradiated levels. The data are derived from at least two experiments for each patient sample, and the standard error of the mean (not shown) is less than 10%.

**Figure 3:**
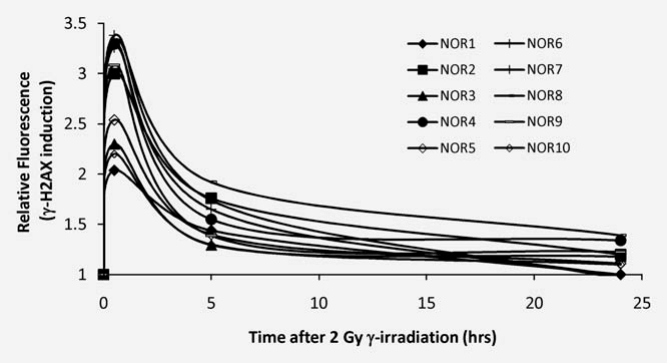
The results derived from the flow cytometric analysis of γ-H2AX induction from ten patients who did not experience excessive NTT (non-overreactor: NOR1–10). A rapid rise in radiation-induced DNA DSB is revealed by an increase in relative fluorescence 30 min postexposure. This is followed by a steady decline in fluorescence, due to repair of DNA DSB and at 24 hr fluorescence levels have returned to near unirradiated levels. The data are derived from at least two experiments for each patient sample, and the standard error of the mean (not shown) is less than 10%.

**Figure 4:**
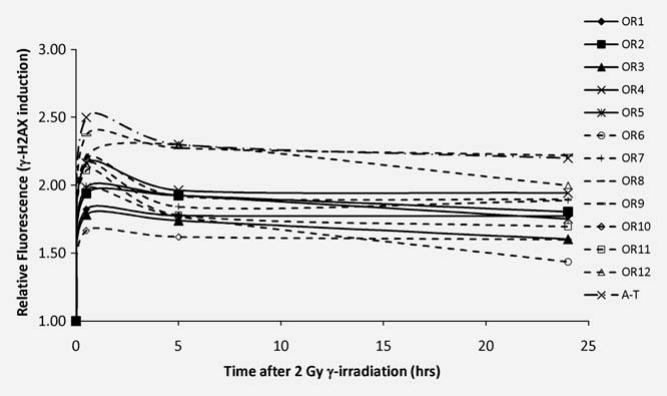
The results derived from the flow cytometric analysis of γ-H2AX induction from 12 cancer patients who experienced severe NTT (overreactor: OR1–12). Patients classified as RTOG4 responders are depicted by an unbroken line (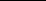) and those classified as RTOG3 are identified by broken lines (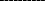). A rapid rise in radiation-induced DNA DSB is revealed by an increase in relative fluorescence 30 min postexposure. Compared to Figure 2, there is retention of fluorescence in the irradiated PBL derived from this group of patients. This retention of fluorescence is consistent with a failure to repair DNA DSB. This is similar to the profile of repair observed in a PBL sample derived from a patient with ataxia telangiectasia (A-T) with a well-established defect in DNA DSB repair (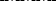). The data are derived from at least two experiments for each patient sample, and the standard error of the mean (not shown) is less than 10%.

Across all groups there is a dramatic induction of fluorescence in PBL samples stained with γ-H2AX antibodies following 2-Gy gamma radiation exposure, representing the induction of DNA DSB. There is then a rapid reduction of fluorescence in PBL samples from healthy noncancer individuals, such that within 5 hr of irradiation levels have fallen to preirradiated control levels as DNA DSB are repaired and the γ-H2AX histone proteins are dephosphorylated (Fig. 2). A similar pattern of damage and timely repair is seen for the NOR group (Fig. 3). However, in those samples taken from OR patients, there is a striking difference with maintenance of the level of fluorescence over a 24 hr period indicating persistent γ-H2AX expression and reduced DNA DSB repair (Fig. 4). For reference, a sample of PBL from a patient with the inherited DNA DSB repair disorder A-T is included, demonstrating very similar results to the OR patients.

**Figure 5:**
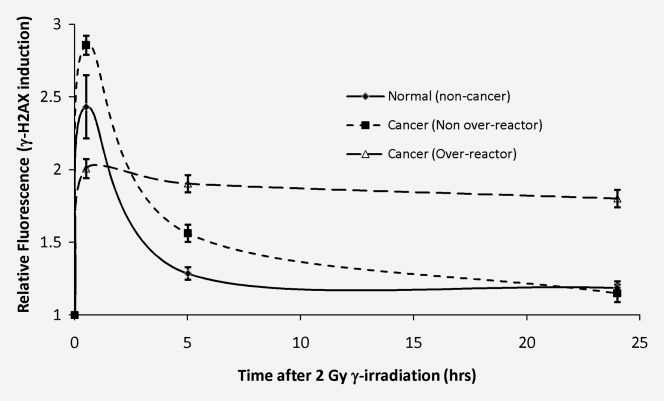
Mean data for each group of patients. The higher levels of fluorescence retention in the patient group that experienced excessive NTT are consistent with the persistence of γ-H2AX in irradiated PBL. This indicates a lack of repair of DNA DSB. However, within the patient group which did not experience abnormal NTT and in the normal (noncancer) group, there is a rapid reduction in fluorescence due to repair of DNA DSB. Error bars represent standard error of the mean.

Figure 5 shows the mean level of induced fluorescence for each group with standard error plotted. Of note, the fluorescence induced at 30 min differs between groups: NOR patients routinely had a higher level of γ-H2AX-associated fluorescence than either OR patients or normal individuals. The level of fluorescence at 24 hr was compared across groups using Student's unpaired *t*-tests: there was significantly higher retention of fluorescence in the OR compared to the NOR group (*p* < 0.0001) and with the N group (*p* < 0.0001). There was no significant difference between the NOR group and the N group (*p* = 0.6).

## Discussion

In our report, we present the results of a study examining whether patients who have suffered excessive toxicity from radiotherapy have impaired normal cell DNA repair mechanisms, with the ultimate aim of developing a diagnostic test to predict such abnormal response. We used flow cytometric analysis of γ-H2AX induction and removal to measure DNA DSB damage and repair in the PBL of cancer patients who had experienced excessive acute and/or late NTT after radiotherapy. These were compared with patients who experienced little or no acute NTT and had developed no significant late NTT within at least 1 year of radiotherapy and with normal noncancer individuals. The γ-H2AX assay was selected for this analysis as it has been shown to be a reliable measure of DNA DSB repair and it is a relatively rapid test from which results can be provided quickly for clinical purposes.^18^, ^19^

We have demonstrated that there is a significant relationship between the persistence of γ-H2AX expression caused by ionising radiation and the level of NTT experienced: at 24 hr there was markedly higher retention of γ-H2AX in PBL from OR patients than NOR or unirradiated normal noncancer control individuals. This finding is of importance for both experimental and clinical oncology as it provides evidence that the γ-H2AX assay when used in a flow cytometric setting may be able to “predict” NTT in cancer patients. Although our results are not strictly predictive as most testing was performed after radiotherapy was delivered, it is certainly plausible that a prospective study could demonstrate that those patients with abnormal persistence of γ-H2AX expression subsequently developed greater than expected toxicity.

Although our assay was able to reliably differentiate between patients clinically classified as NOR or OR patients, we could not differentiate between those individuals with the severest NTT (RTOG 4) and those with moderately severe NTT (RTOG 3). This lack of apparent sensitivity should not, however, be seen as a failing of the test as there are a great many factors involved in the development of toxicity, not least the variation between different clinical observers in scoring toxicity grade. Other factors include and are not limited to total dose, dose per fraction, duration of treatment, nature of normal tissue irradiated, whether acute or late toxicity is assessed (owing to different α/β ratios) and exposure to chemotherapy, either neoadjuvant or concomitant or other pharmaceutical modifiers. A further study with a larger patient cohort attempting to control for such factors may provide useful results, especially if coupled with more refined and sensitive flow cytometry such as the Imagestream (Cronus, Surrey, UK) in which DNA DSB can be quantified at the individual cell level.

However, a predictive test would be of most clinical benefit in being able to detect those (relatively rare) individuals at risk of severe toxicity far greater than would be expected for a proposed treatment plan; for example, individuals who may suffer severe long-term consequences of treatment—as in the carefully selected OR group here—or even fatal complications as have been noted above. If the predictive test indicated that radiotherapy had a very high probability of causing severe NTT, an alternative strategy could be entertained, such as surgery for primary treatment, or omission of radiotherapy in the adjuvant setting. If radiotherapy was not contraindicated but still thought likely to cause notable toxicity, the opportunity would arise to tailor treatment to the patient, such as by offering intensity-modulated radiotherapy to minimise the regions of higher dose to uninvolved organs at risk. Our results strongly suggest that the γ-H2AX assay has merit as a predictor of excessive toxicity. Although it may not be feasible to offer the test to every patient embarking upon a course of radical radiotherapy, a targeted approach to patients thought to have a risk of impaired DNA repair, such as those with a strong family history of malignancy, known repair defect such as *BRCA1* or *BRCA2* or even excessive clinical reaction to neoadjuvant chemotherapy, would be a practical alternative. It has been demonstrated that *BRCA1* patients are hypersensitive to DNA-damaging cytotoxic chemotherapy,^20^ and there may also be scope for using the γ-H2AX assay to predict response to chemotherapy.

Interestingly, we observed that there are significantly different levels of fluorescence induced at 30 min after 2-Gy gamma exposure between the groups of individuals. The highest levels of fluorescence were observed in the ten NOR patients, and the lowest levels of damage induction were paradoxically observed in PBL derived from OR patients, even though these cells failed to effectively repair the DNA DSB as measured in this assay. The reasons for these observations remain unclear. One explanation may be the reported observation of significant interindividual differences in the induction of γ-H2AX when measured with flow cytometry.^21^, ^22^ However, it is difficult to accept this as the sole explanation because the difference between groups is so marked: where this to be the only cause one would expect more of an overlap between groups owing to an averaging of differences between individuals across groups. The question is raised as to whether OR patients are not only less able to repair DNA damage in response to irradiation but perhaps less able to detect such damage, demonstrated by the relatively lower induction of γ-H2AX. Further research would provide valuable insight into this postulation.

Although we have found a strong correlation between γ-H2AX persistence and the development of excessive acute or late NTT, other studies have not demonstrated such a strong relationship. Werbrouck *et al*. observed no differences in kinetics of γ-H2AX foci following irradiation of PBL from 29 patients with gynaecological malignancies who developed differing levels of NTT.^23^ Vasireddy *et al*. examined γ-H2AX induction in 18 patients with acute and late NTT classified as RTOG 3 and saw similar levels of γ-H2AX induction and removal over a 24 hr period for the majority of patients and controls.^24^ However, these authors were in fact able to detect a patient with defective DNA repair mechanisms and suggest that determining γ-H2AX levels in irradiated PBL may contribute to identifying individual radiosensitivity. The discordance between these observations and ours may well reflect the fact that individuals with DNA repair defects are rare in the general, or even cancer patient, population. Any prospective study is unlikely to encounter many such patients unless including very large numbers; our study targeted ORs identified on clinical grounds and compared them with known normal reactors, thus greatly increasing the likelihood of detecting a true difference.

In summary, our study has demonstrated cancer patients developing severe NTT fail to repair radiation-induced DNA DSB as efficiently as either patients experiencing little or no NTT and normal noncancer individuals although it does not probe the mechanism of this inefficiency. It is an interesting question to speculate whether the OR patients described here may harbour subtle hypomorphic mutations in key DNA repair genes involved in the recognition and processing of DNA DSB. Previous studies have demonstrated that individuals with mutations in DNA DSB repair genes exhibit extreme clinical and cellular hypersensitivity to radiation, and further study of our patient cohort may yet reveal such mutations. From a clinical standpoint, our study provides the exciting prospect that a clinician may be able to predict and so avoid the overresponse of patients to radiotherapy before a single dose has been delivered.
